# From Binary to Ternary Transition-Metal Nitrides: A Boost toward Nitrogen Magneto-Ionics

**DOI:** 10.1021/acsami.2c12847

**Published:** 2022-09-21

**Authors:** Zhengwei Tan, Sofia Martins, Michael Escobar, Julius de Rojas, Fatima Ibrahim, Mairbek Chshiev, Alberto Quintana, Aitor Lopeandia, José L. Costa-Krämer, Enric Menéndez, Jordi Sort

**Affiliations:** † Departament de Física, Universitat Autònoma de Barcelona, E-08193 Cerdanyola del Vallès, Spain; ‡ Department of Physics, Durham University, South Road, DH1 3LE Durham, U.K.; § University of Grenoble Alpes, CEA, CNRS, SPINTEC, 38000 Grenoble, France; ∥ Institut Universitaire de France, 75231 Paris, France; ⊥ Institut de Ciència de Materials de Barcelona (ICMAB-CSIC), Campus UAB, Bellaterra, E-08193 Barcelona, Spain; # Catalan Institute of Nanoscience and Nanotechnology (ICN2), CSIC and BIST, Campus UAB, Cerdanyola del Vallès, E-08193 Barcelona, Spain; ¶ IMN-Instituto de Micro y Nanotecnología (CNM-CSIC), Isaac Newton 8, PTM, 28760 Tres Cantos, Madrid, Spain; ∇ Institució Catalana de Recerca i Estudis Avançats (ICREA), Pg. Lluís Companys 23, E-08010 Barcelona, Spain

**Keywords:** magnetoelectricity, voltage control of magnetism (VCM), magneto-ionics, transition metal nitride, ion diffusion

## Abstract

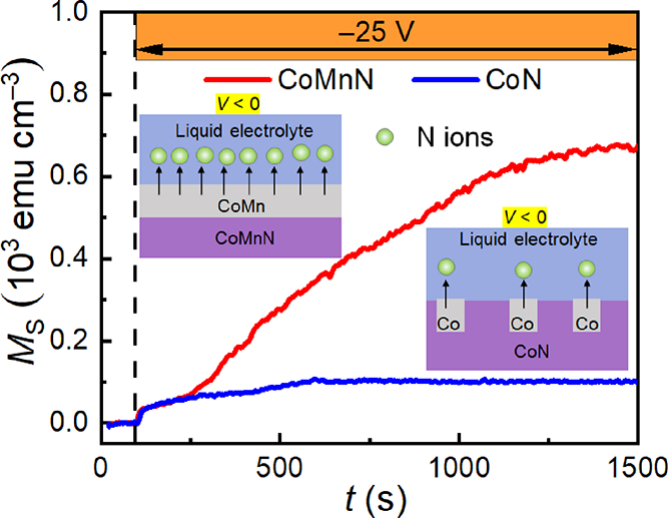

Magneto-ionics is an emerging actuation mechanism to control the magnetic properties of materials *via* voltage-driven ion motion. This effect largely relies on the strength and penetration of the induced electric field into the target material, the amount of generated ion transport pathways, and the ionic mobility inside the magnetic media. Optimizing all these factors in a simple way is a huge challenge, although highly desirable for technological applications. Here, we demonstrate that the introduction of suitable transition-metal elements to binary nitride compounds can drastically boost magneto-ionics. More specifically, we show that the attained magneto-ionic effects in CoN films (*i.e.*, saturation magnetization, toggling speeds, and cyclability) can be drastically enhanced through 10% substitution of Co by Mn in the thin-film composition. Incorporation of Mn leads to transformation from nanocrystalline into amorphous-like structures, as well as from metallic to semiconducting behaviors, resulting in an increase of N-ion transport channels. *Ab initio* calculations reveal a lower energy barrier for CoMn–N compared to Co–N that provides a fundamental understanding of the crucial role of Mn addition in the voltage-driven magnetic effects. These results constitute an important step forward toward enhanced voltage control of magnetism *via* electric field-driven ion motion.

## Introduction

The intentional incorporation of impurities into a host material, a process known as element substitution,^1^ is of fundamental significance in reconstructing nanocrystals,^2^ modifying electronic characteristics,^3^ or modulating magnetism,^4,5^ among others. Controlled element introduction has been proved effective in yielding semiconductor-based hybrid materials with desirable properties for target applications, such as solar cells,^6^ batteries,^7^ novel transistors,^8,9^ superconductors,^10^ or spintronics devices.^4,5,11,12^ Recently, unprecedented control of magnetic properties (including “ON–OFF” switching of ferromagnetic states^13^) has been realized using magneto-ionic actuation.^14^ This is an emerging energy-efficient approach to tune magnetism based on electric field-induced ions (*e.g.*, O^2–^,^13−17^ Li^+^,^18^ F^–^,^19^ H^+^,^20−22^ or N^3–^^23−25^) motion. However, element introduction in magneto-ionic systems, that may allow for further engineering of magnetic properties with voltage, is still unexplored.

A typical magneto-ionic system comprises the target material, usually a ferromagnetic (FM) metal or an oxide,^26^ and a solid or liquid electrolyte (adjacent to the target material), working as dielectric and ion reservoir layers. One of the most studied systems is Co/GdOx, in which control of the perpendicular magnetic anisotropy through voltage-driven oxygen migration has been demonstrated.^14^ One problem with O^2–^ transport-based magneto-ionics at room temperature is its poor endurance, owing to slow and irreversible chemical/structural changes that occur when voltage polarity is reversed.^15^ Smaller ions, such as H^+^, F^–^, or Li^+^, have been utilized to achieve faster and more cyclable voltage-induced changes of the magnetic properties. However, these systems pose some limitations in terms of compatibility with traditional complementary metal-oxide semiconductor (CMOS)-based devices.^27^ Alternatively, nitrogen magneto-ionics have been demonstrated by using a cobalt nitride target material (*i.e.*, CoN^23^), which provides “ready-prepared” lattice sites for ionic diffusion, leading to improved endurance at room temperature, without sacrificing operating speed and compatibility. In addition, the microstructure of the magneto-ionic target materials play an important role in the attained values of resistivity and ion transport mechanisms, resulting in a planar-like migration front for ions,^24,25^ that is highly appealing for devices when compared to traditional inhomogeneous or cross-sectional ion diffusion channels typically occurring upon O^2–^-ion migration.^13,16^ Magneto-ionics is essentially a dynamic process,^17^ which involves breaking and recombining metal-O or metal-N bonds. This approach requires an effective electric field to drive the process, as well as properly tuned magnetic materials to be modulated.^28^ Although ferromagnetic metals show large and stable magnetization, high *T*_C_, and, eventually, a well-defined perpendicular magnetic anisotropy,^29^ their direct utilization for magneto-ionics also poses some drawbacks. Among those is the limited electric-field screening length stemming from their high electric conductivity, which means that the effects of voltage are strongly limited to the outer surface of the metal.^30^ This limitation can be overcome, to some extent, using semiconductors instead of metals.^29^ Thus, if semiconducting properties could be imparted to target materials without sacrificing their magnetic properties, magneto-ionic performance may be potentially boosted. To achieve this goal, introduction of certain elements leading to precise structural and compositional engineering at the nanoscale, together with highly tunable magnetic and electrical properties, may turn out to be a suitable strategy. Finding a suitable element and introducing it in a controlled manner into a host FM target material is thus critical. In metallic alloys, efforts have been made to substitute Co, Ni, or Fe by Mn as an effective means to alter their magnetic and electrical behavior.^31−34^ In magneto-ionics, Mn introduction may also give specific advantages. First, Mn, as an “amorphizing” agent,^35^ could favor the formation of an amorphous phase, which would promote planar-like diffusion fronts for ion motion (because, otherwise, ion motion preferentially occurs along grain boundaries). Also, Mn-substitution could introduce more defects in the crystal lattice and increase electrical resistivity, possibly leading to the modification of electrical properties, from metallic-like to semi-metallic or semiconducting behavior.^33^ Incorporation of Mn may trigger hopping mechanisms and hence change the transport properties, as studied in earlier cobalt ferrite systems.^33,34^ Most importantly, the enhancement of saturation magnetization and perpendicular magnetic anisotropy of host FM target materials with moderate Mn introduction has also been confirmed both from experimental and theoretical studies.^36−38^ Therefore, introducing Mn into FM nitrides (*e.g.*, CoN) might result in synergetic effects and could make magneto-ionics more attractive for spin-based and other magnetoelectric devices.

In this work, we show multiple benefits that moderate substitution of Co by Mn brings to the magneto-ionic performance of CoN-based heterostructures. With the addition of Mn, a change from nanocrystalline to amorphous structures is observed, accompanied by a transition from metallic-like to semiconducting properties. The former increases N-ion transport channels, as verified by high-angle annular dark-field scanning transmission electron microscopy (HAADF-STEM) and electron energy loss spectroscopy (EELS). The latter significantly extends the electric field effect that is normally limited to a few Å in metals.^30^ Mn incorporation leads to a 6.7-fold voltage-driven enhancement of the saturation magnetization (*M*_S_) and improved high-frequency cyclability. In addition, *ab initio* calculations have been performed to estimate the Co–N and CoMn–N energy barriers using different crystal orientations and N atom insertion paths. In all cases, a reduced energy barrier for CoMnN is obtained, compared to CoN. These experimental and theoretical findings prove the highly beneficial effect of Mn-substitution in enhancing magneto-ionics. These results could have implications in diverse technological areas, like neuromorphics^39,40^ or iontronics in general.

## Results and Discussion

Cobalt nitride (CoN) and cobalt manganese nitride (Co_0.9_Mn_0.1_N, identified as CoMnN throughout the text), both 30 or 100 nm thick, were grown on Cu (10 nm)/Ti (10 nm)/[100]-oriented Si substrates by reactive magnetron sputtering (see the Methods section).

**Figure 1:**
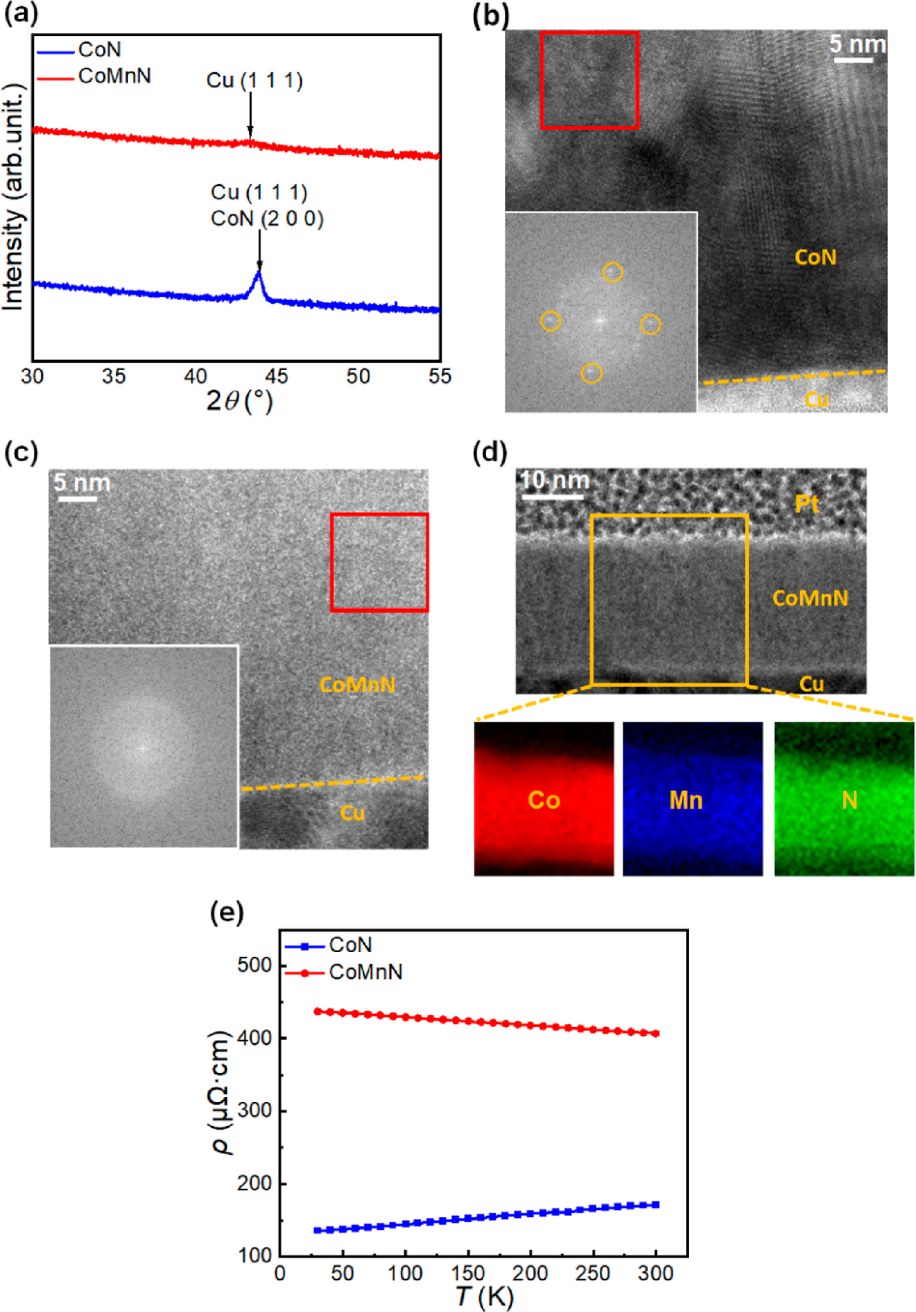
Structural, compositional, and electric transport characterization of as-deposited films. (a) θ/2θ XRD patterns of the as-prepared 100 nm thick CoN and CoMnN films. (b,c) High-resolution TEM images of the cross section of 30 nm thick CoN and CoMnN films. The inset shows the fast Fourier transform of the area marked with a red square. (d) HAADF-STEM micrograph and EELS Co, Mn, and N elemental mappings for as-prepared 30 nm thick CoMnN films. (e) Electrical resistivity (ρ) measured as a function of temperature from 30 to 300 K, for as-deposited 30 nm thick CoN and CoMnN films.

Figure 1a shows the X-ray diffraction (XRD) patterns of 100 nm thick CoN and CoMnN films. A single diffraction peak is observed for the CoN film at a 2θ ≈ 43.84°, which is consistent with both (111) Cu (JCPDF card no. 00-001-1241) or expanded (200) CoN (JCPDF card no. 00-016-0116) phases. In contrast to CoN, the CoMnN film does not show such well-defined peaks but only a broad and weak peak centered at 2θ ≈ 43.28°, which is also consistent with (111) Cu or expanded (200) CoMnN. However, CoMnN clearly exhibits a much lower crystallite size value, evidencing the nanostructuring effect caused by Mn. Because the nitride films are 10 times thicker than the Cu buffer layer, the signal arising from both patterns is mainly attributed to the CoN and CoMnN phases. Both films are textured along (200) planes and nanocrystalline. In the case of the CoMnN, the degree of nanostructuring is more pronounced, approaching a highly disordered (eventually amorphous-like) structure.

This result is confirmed by high-resolution transmission electron microscopy (HRTEM) images of the cross-sections of as-prepared CoN and CoMnN films, as demonstrated in Figure 1b,c, respectively. The areas marked with red squares were chosen for fast Fourier transform (FFT) analyses, as shown in the insets, in which clear diffraction spots can be found for CoN films [points inside yellow cycles, corresponding to an interplanar distance *d* = 2.13 Å, consistent with a cubic CoN (200) orientation], whereas no spot is observed for CoMnN films, in agreement with XRD results and evidencing the role of Mn as a “amorphizing” agent.^35^ To further understand the compositional distribution of CoMnN films, HAADF-STEM and EELS were performed on as-deposited CoMnN films, with corresponding mappings shown in Figure 1d. The Co (red), Mn (blue), and N (green) elements are all uniformly distributed in the films, evidencing the homogeneous growth process of CoMnN. An amorphous or very nanocrystalline structure should promote the formation of a larger density of ion diffusion channels under the application of an applied voltage, compared to a well-crystallized structure with limited amounts of grain boundaries, eventually allowing for a greater modulation of magnetic properties with an electric field.^23,24^

To investigate the role of Mn on electric transport, resistivity (ρ) as a function of temperature (*T*, in the range 30–300 K) was measured on both as-prepared CoN and CoMnN films. As seen in Figure 1e, the resistivity at room temperature (300 K) is approximately 172 μΩ cm for CoN films (the value is close to that of metallic Co^41^ and previously reported less-stoichiometric Co-rich CoN films prepared by a triode system^25^) and about 406 μΩ cm for CoMnN films. Moreover, the resistivity of CoN is observed to monotonically increase (dρ/d*T* > 0) throughout the whole measured temperature range, which is consistent with metallic behavior.^42^ This metallic behavior has also been reported in other transition-metal nitrides,^43^ and it is attributed to some degree of wavefunction overlap. In CoN, electrons are localized and there is a little overlap between the wavefunction of ions situated on adjacent sites.^44^ In contrast, the CoMnN films show a monotonic decrease (dρ/d*T* < 0), corresponding to semiconducting behavior,^45^ due to the increase in drift mobility of charge carriers at higher temperatures. This result demonstrates that partial substitution of Co by Mn not only increases the resistivity but also turns the film semiconductor. In addition, the variation of carrier densities (n/p) as a function of temperature for CoMnN (Figure S1) reveals an overall negative value (by convention meaning predominant electron transport, as opposed to positive when holes would dominate), hence showing that introducing Mn makes electrons the dominant charge carriers while changing the behavior from metallic-like to semiconductor (Figure 1d). Additionally, the resistivities of N-free Co and CoMn films (Co_0.9_Mn_0.1_) were measured to be 100 and 470 μΩ cm, respectively. Thus, Mn incorporation increases ρ both for N-free and the nitride films. While the introduction of Mn plays a role like increasing the nitrogen concentration in the films in terms of electrical properties, the interplay between nitrogen content and defects on resistivity still remains to be fully elucidated. Namely, the degree of bonding with nitrogen, effects of variable amounts of introduced Mn, possible charge compensation effects and impurity scattering in these polycrystalline films need to be further studied. Anyhow, the increased resistivity and thus the change of electric behavior from metallic to semiconducting achieved by the addition of Mn enables a deeper penetration of the electric field (Figure S2), up to few tens of nm,^25^ enhancing magneto-ionic effects, as will be shown below.

**Figure 2:**
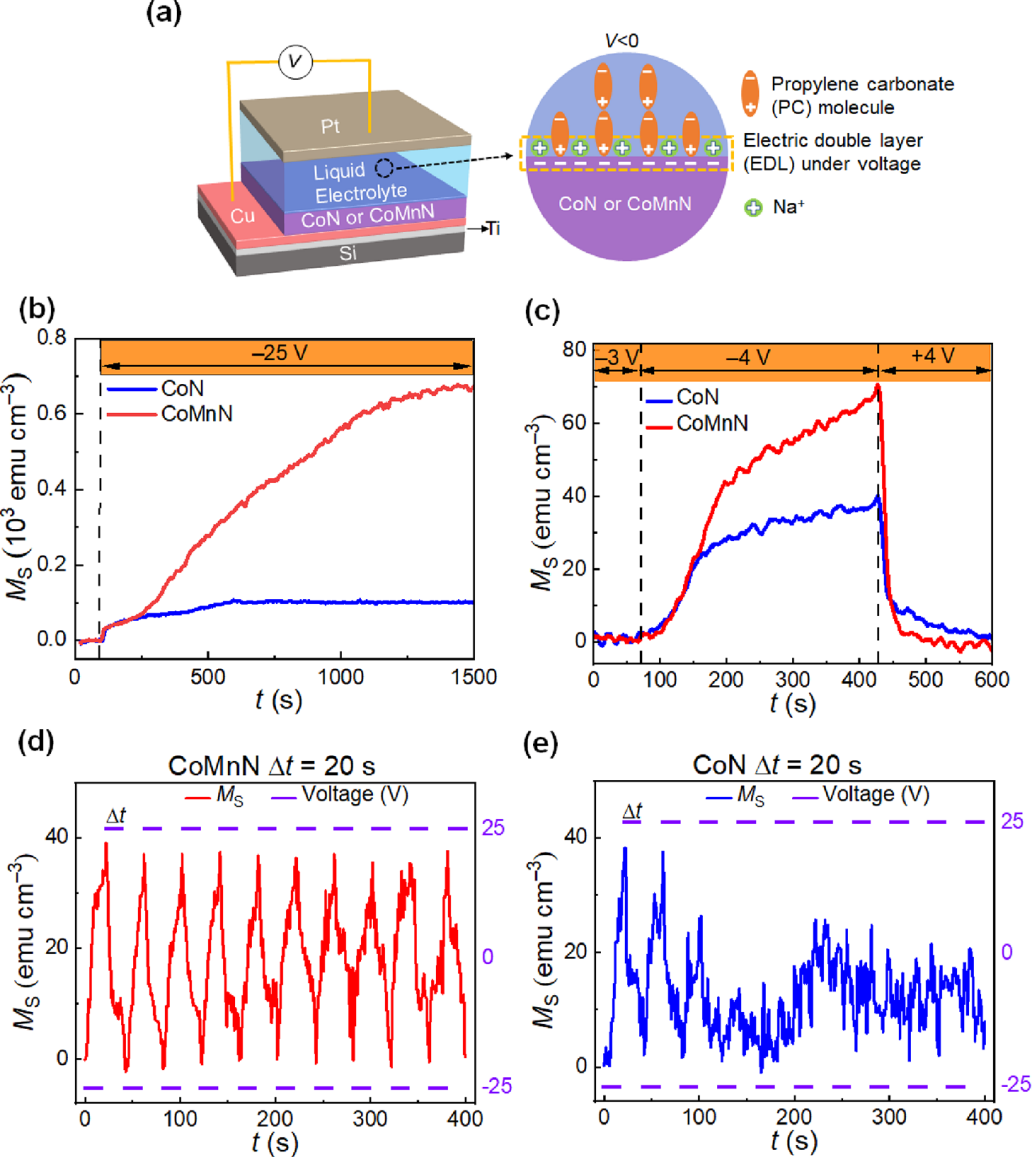
Magneto-ionic characterization of 30 nm thick CoN and CoMnN films under electrolyte gating. (a) Schematic of the designed structure for electrolyte actuation (left) and sketch of the formation of electric double layer during voltage actuation (right). (b) Time (*t*) evolution of the saturation magnetization *M*_S_ for CoN and CoMnN films under −25 V. (c) *t* evolution of *M*_S_ when the voltage is monotonically increased in steps of −1 V to determine the onset voltage required to trigger magneto-ionics in CoN and CoMnN films. (d,e) Magneto-ionic cyclability of the CoMnN and CoN films subjected to −25 V/+25 V voltage pulses applied with a periodicity of 20 s.

To investigate the effect of Mn introduction on the magneto-ionic performance, both 30 nm thick CoN and CoMnN thin films were electrolyte-gated in a capacitor-like configuration using a Pt wire as the counter electrode, while performing in-plane magnetic measurements by vibrating sample magnetometry (VSM) at room temperature. Propylene carbonate with Na^+^- and OH^–^-solvated species was employed as an aprotic, anhydrous polar liquid electrolyte to serve as a nitrogen-ion reservoir^23−25^ and to apply a uniformly distributed, out-of-plane electric field through the formation of an electric double layer (EDL)^30,46^ at the films’ upper surface (as shown in Figure 2a).

As seen in Figure S3a, the as-prepared CoN sample exhibits some traces of ferromagnetic signal (<21 emu cm^–3^, which is equivalent to ≈1.5% the magnetization of pure Co^47^), whereas the as-prepared CoMnN sample shows a virtually paramagnetic behavior (<3 emu cm^–3^). This reduction of residual ferromagnetism in the as-prepared sample by addition of Mn is possibly the result of the reported difference of cohesive energy between Mn–N and Cu–N (≈1.17 eV/atom) being smaller than that between Co–N and Cu–N (≈2.01 eV/atom).^48^ This means that the tendency to form off-stoichiometric regions (*i.e.*, Co-rich CoN clusters) near the CoN/Cu(N) interface is slightly larger than for CoMnN/Cu(N).

Figure 2b shows the variation of *M*_S_ of CoN and CoMnN films as a function of time (*t*) during electrolyte gating at −25 V, while an external magnetic field of 10 kOe (above the anisotropy field of the generated ferromagnetic counterpart) was applied to ensure magnetic saturation. In response to the applied voltage, an immediate increase of *M*_S_ is observed in both CoN and CoMnN films, indicating a quick onset for nitrogen motion, resulting in the appearance of metallic Co(Mn) ferromagnetic phase.^23^ Interestingly, while CoN and CoMnN show similar increasing trends of *M*_S_ during the initial stages of voltage actuation (*i.e.*, the first 150 s), *M*_S_ subsequently levels off for CoN films but continues to increase in CoMnN films before reaching a stable value for times close to 1500 s. As a result, a 6.7-fold enhancement in the obtained steady-state value of *M*_S_ is achieved in voltage-actuated CoMnN compared to CoN, from 102 to 672 emu cm^–3^, respectively (in agreement with the magnetic hysteresis loops shown in Figure S3b,c). Note that, as reported earlier,^37,38^*M*_S_ for CoMn is slightly larger than for pure Co (Figure S3d), but this fact by itself alone cannot explain the obvious magneto-ionic enhancement of *M*_S_ observed in their nitrides and demonstrates that Mn-substitution boosts magneto-ionics by some other mechanisms, for example, the dissimilar resistivity variations during the denitrification processes in CoN and CoMnN films. With the goal to investigate the eventual influence of the growing method and to be able to compare with previously reported results, 85 nm thick CoN and CoMnN films were also grown by triode sputtering. This is the thickness of the CoN films investigated in our previous works.^23−25^ As shown in Figure S4, the incorporation of Mn also results in a larger generation of magnetization analogously to what happens when comparing the magneto-ionic response of CoN and CoMnN films grown by magnetron sputtering (Figure 2b). It is remarkable that, even though the films grown by triode sputtering are thicker than those prepared by magnetron sputtering (85 nm *vs* 30 nm, respectively), larger magnetization values are attained upon voltage applications in films grown by triode sputtering, both in the CoN and CoMnN systems. This suggests the importance of a more nanocrystalline microstructure obtained by triode sputtering in the enhanced nitrogen-ion motion due typically to a larger defect size and density. In any case, our results confirm the role of Mn as an “amorphizing” agent in improving magneto-ionics, regardless of the growing method.

The minimum threshold voltage required to trigger magneto-ionic effects is of large importance for device implementation.^24^ Thus, the onset voltages were also evaluated in both systems by monotonically increasing the external voltage in steps of −1 V until *M*_S_ started to increase, as shown in Figure 2c. The obtained results reveal that the onset voltage is approximately −4 V for both CoN and CoMnN films. Figure 2c also shows that, in spite of a larger *M*_S_, the recovery time for CoMnN films is significantly shorter than for CoN films, thereby a better endurance in the case of CoMnN films is envisaged. Taking this into account, the cyclability was investigated by subjecting the CoN and CoMnN films to −25 V/+25 V pulses with a periodicity of 20 s. As shown in Figure 2d, a very stable and reversible cycling behavior for CoMnN is obtained, whereas no signs of magneto-ionic effects are continued after two cycles for CoN films (Figure 2e). Nonetheless, for longer pulse duration (with voltage switched every 2 min), CoN films show a relatively stable cyclability as well (Figure S3e). This demonstrates that Mn-substitution is not only favorable to enhance the magneto-ionic rates or the attained *M*_S_ values but also the cyclability (or endurance) through improvement of the recovery process.

**Figure 3:**
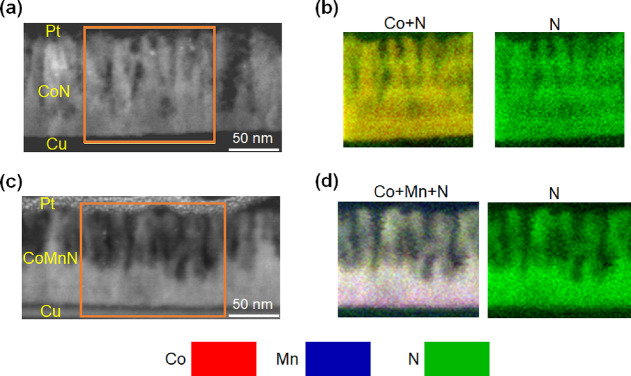
Compositional characterization by HAADF-STEM and EELS of the magnetoelectrically actuated films. HAADF-STEM and elemental EELS mappings corresponding to areas marked in orange for 100 nm thick (a,b) CoN and (c,d) CoMnN films gated at −25 V for 40 min, respectively. Cu and Pt layers serve as working electrodes and protective capping layer for lamellae preparation, respectively. Colors corresponding to each element are noted at the bottom of the figure, that is, Co, Mn, and N are represented by red, blue, and green colors in the EELS elemental mappings, respectively.

To further understand the effect of Mn substitution from the perspective of voltage-driven ion transport, cross-sectional lamellae of 100 nm thick CoN and CoMnN films electrolyte-gated at −25 V for 40 min were studied by HAADF-STEM and EELS. As shown in Figure 3a, a moderate denitriding process occurs in CoN, which results in sparse, cross-sectional (*i.e.*, perpendicular-to-film) channels after nitrogen-ion diffusion has taken place, similar to what was reported in other magneto-ionic systems like Co_3_O_4_ also grown by magnetron sputtering.^13,16^ This columnar morphology suggests that N-ion motion takes place, at least to some extent, along these channels generated perpendicular to the film plane during voltage applications. As shown in Figure 3b, EELS mappings reveal a lack of N and depletion of Co in the channels, in accordance with the formation nanoporosity in the films (in the form of elongated vertical pore channels of 5–10 nm in width). The formation of these channels in the upper part of the films is even more evident for the magneto-ionically treated CoMnN films (see Figure 3c,d). The formation of such channels can have an influence on both the electric properties (electronic hopping) as well as the time-dependence evolution of magneto-ionic effects, although it is quite difficult to establish the exact current flow paths in this kind of nanocrystalline materials. Remarkably, in this case, a planar horizontal front, separating porous and denser areas, is observed. The occurrence of such planar diffusion fronts was previously reported in CoN films grown by triode sputtering (not by magnetron sputtering as in the current work) exhibiting a highly nanocrystalline microstructure and larger generated magnetization.^23,24^ Remarkably, the presence of metallic Co was confirmed by the fast Fourier transform (FFT) spots obtained from high-resolution TEM, as shown in Figure S5, which is responsible for the *M*_S_ increase after voltage treatment (Figure 2b). These observations are consistent with vertical and horizontal line profiles of the N element, as shown in Figure S6, which reveal an obvious difference in N content between the upper and bottom parts of the CoMnN films (in contrast to CoN, where the N concentration is more homogeneous). Additionally, the nanoporosity causes sudden jumps of the N content along the horizontal profiles for both CoN and CoMnN films. These results reveal that, under the same magnetoelectric actuation conditions, a larger amount of nitrogen is released to the electrolyte in CoMnN compared to CoN films. This agrees with Figure 2b,c, which show a larger increase of *M*_S_ in CoMnN. Overall, the HAADF-STEM and EELS observations on CoN and CoMnN films illustrate that Mn substitution significantly increases nitrogen transport channels, leading to an enhanced magneto-ionic behavior (thus explaining the shorter recovery time and the improved cyclability).

**Figure 4:**
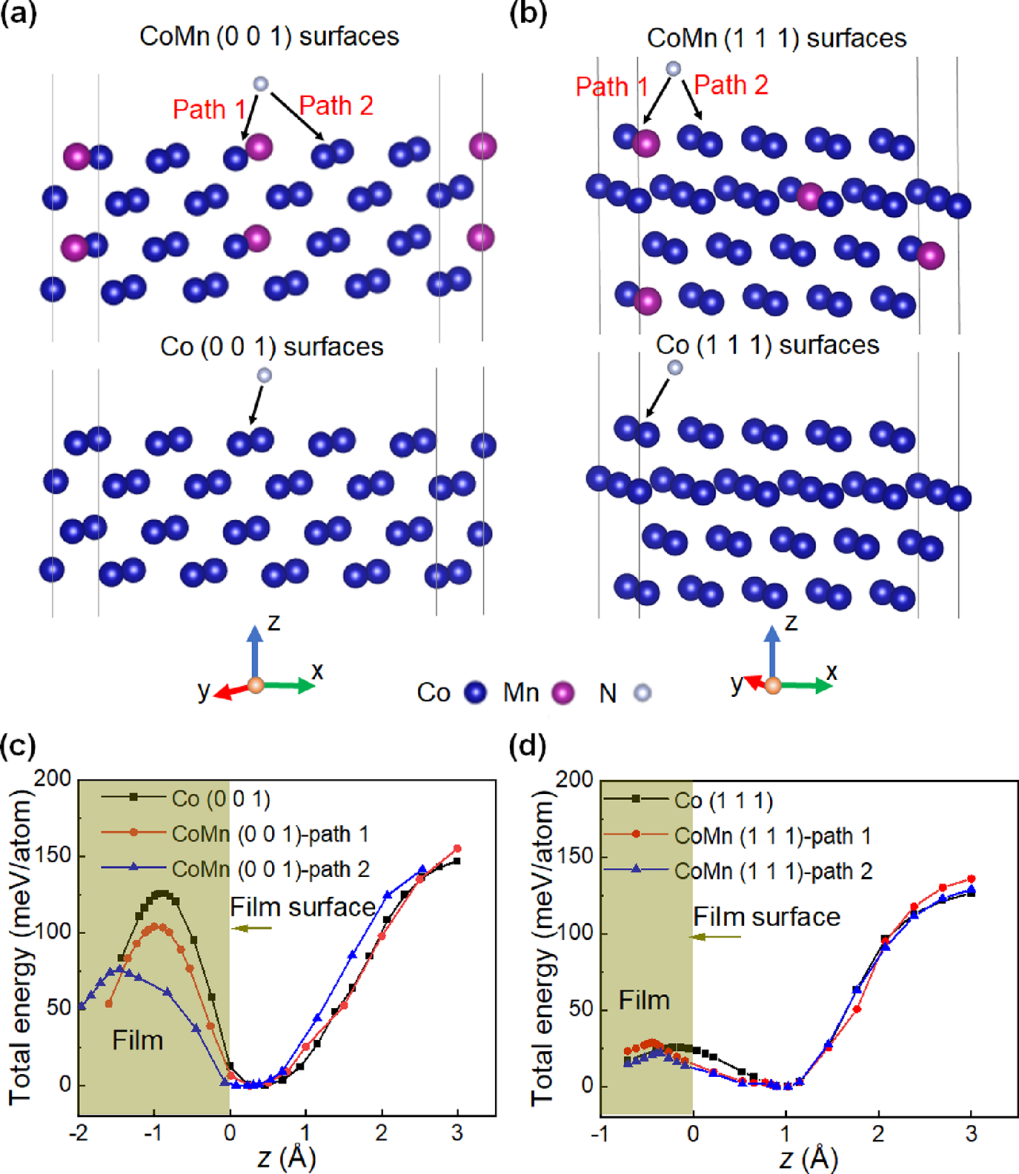
*Ab initio* calculations of Co–N and CoMn–N energy barriers. Schematic of (a) CoMn and Co (001) surfaces and (b) CoMnN and CoN (111) surfaces, respectively, designed for N-atom insertion. For CoMnN surfaces, both Co–Mn path (path 1) and Co–Co path (path 2) have been considered. (c,d) Total energy per atom as a function of distance for the aforementioned surfaces. The total energy is plotted relative to the minimum energy values, as a function of the displacement between the reference CoMn or Co outermost surface atom and the inserted N atom.

The efficiency of magneto-ionics initially depends on the minimum energy required for breaking and re-forming magnetic metal-ion bonds. Using the nudged elastic band method (NEB)^49,50^ (see the Methods section), the Co–N and CoMn–N energy barriers were calculated in a case where a nitrogen ion is inserted into a cobalt or cobalt manganese surface with 10 atoms per layer (Figure 4a,b). Two orientations, (001) and (111), were considered and the total energies per atom were calculated as a function of the distance z between the inserted N atom and a Co or CoMn upper surface (see Figure 4c,d, respectively). Furthermore, for the case of CoMnN films, the formation energy of the N insertion into either Co–Mn (path 1) or Co–Co (path 2) were also calculated. The position *z* = 0 Å refers to the outmost layer of Co or CoMn, while its negative sign represents the displacement inside of the film (and vice versa for the positive sign). For the Co (001) surface, CoMn (001) surface-path 1 and CoMn (001) surface-path 2, the local minimum (maximum) energies are located at around *z* = 0.4 (−1 Å), 0.4 (−1 Å), and 0.3 (−1.4 Å), respectively, with corresponding energy barriers between the two extrema are calculated to be 126, 104, and 76 meV/atom. Further calculation shows that the energy barrier needed to be overcome by N is lower for the CoMn (001) surface (by 22 meV/atom for path 1 and 50 meV/atom for path 2) compared with the Co (001) surface. This reduction of energy barrier by Mn addition is consistent with the larger quantity of channels and nanoporosity due to N-ion movement observed in CoMnN compared to CoN films, which brings about a larger *M*_S_ and faster recovery process (thus improved cyclability) for CoMnN films.

The results for the (111) surfaces, however, are more complex. When a N atom is introduced into the Co surface, the positions of local minimum and maximum energies are *z* = 1.1 and −0.1 Å, respectively, while the corresponding positions for the CoMn-path 1 case are *z* = 1.1 and −0.4 Å. In turn, the calculated energy barriers between the two extrema are 25.4 and 28.9 meV/atom for nitrogen displacement in Co and CoMn-path 1 structures, respectively. Note that we find a reduced barrier height for Co–N in the present study compared to ref (23). This is explained by the dependence of the energy barrier on the concentration of the inserted atoms into a surface. In ref (23), the ratio of N to Co atoms per surface layer was 1:1 and this implies the 100% concentration of N insertion into the surface, whereas here we have a supercell, which is laterally larger to simulate a 10% N insertion into the surface. When comparing Co and CoMn-path 1 structures, the minimum energy required by N to enter the CoMn (111) orientation is slightly larger than for Co (111). However, further energy barrier calculation for CoMnN-path 2 structure gives a value of 21.8 meV for (111) interfaces, which is lower than that obtained in case of path 1. Therefore, overall, the differences in energy barriers between Co and CoMn along this lattice orientation are less evident than along the (001) orientation, and they are path dependent. In fact, the considered N paths in the calculations are particular, while in the experiment not all N atoms have essentially the same path into the surface and thus a collective effect of perhaps several paths is observed. Also, it is instructive to comment on the difference in the positions of the minima/maxima in Figure 4c,d. We believe that it is caused by the different crystallographic orientations. For the (111)-oriented surface, the maximum energy is reached when the N atom is almost located in the first atomic surface layer. However, in the (001) surface, the maximum energy is found when the N atom is integrated more into the surface. In this case, it feels less comfortable due to smaller interlayer spacing to accommodate it, and thus the barrier is larger. Remarkably, these calculations show that the lattice orientation plays a key role in the observed variations in energy barriers upon Mn-substitution, which provides a valuable guide for precisely engineering magneto-ionics from a structural perspective.

## Conclusions

In summary, here we demonstrate that magneto-ionic effects in CoN films are drastically enhanced by the introduction of Mn (in low percentages) to the binary composition of the transition metal nitride. Such enhancement is ascribed to the key role of Mn in modifying the microstructure and electric transport properties of the CoN films, as well as the change in the formation energy of the metal-ion bonds. The incorporation of Mn was proven effective to bring a more amorphous microstructure and semiconducting properties to CoN. As a result, a 6.7-fold enhancement of the saturation magnetization and higher magneto-ionic cyclability are achieved. From *ab initio* calculations, the energy barrier in CoMn–N is overall smaller than for Co–N, providing hints to understand the more efficient N-ion motion. The reported enhanced ion motion effects in CoMnN films by moderate Mn introduction (and the extension to other transition-metal systems) are appealing for diverse technological areas (beyond magneto-ionics), such as electrochemical catalysis, batteries, solar cells, or spintronics.

## Methods

### Sample Fabrication

CoN and Co_0.9_Mn_0.1_N thin films with thicknesses of 30 nm or 100 nm were grown at room temperature by magnetron sputtering in a high-vacuum chamber (with a base pressure <8 × 10^–8^ Torr) on non-doped (100)-oriented Si wafers (0.5 mm thick) previously coated with a 10 nm thick titanium adhesion layer and 10 nm thick copper seed layer. The copper was partly masked to avoid the nitride film deposition and later serve as working electrodes. Pure Co and Mn targets were co-sputtered for the deposition of CoMnN and rates were calibrated to obtain a Co/Mn ratio of 9:1. The growth of CoN and CoMnN was carried out in a mixed Ar and N_2_ atmospheres always using a nitrogen partial pressure of 50% and a total working pressure of 3 × 10^–3^ Torr. The distance between the substrate and targets was around 10 cm, and the sputtering rate was approximately 0.8 Å s^–1^.

### Magneto-Ionic Characterization

*In situ* magnetoelectric measurements were carried out at room temperature using a commercial vibrating sample magnetometer (VSM) from Micro Sense (LOT, Quantum Design), with a maximum applied magnetic field of 2 T. Electrolyte gating was conducted between the counter electrode (a Pt wire) and the working electrode (the investigated CoN/Cu/Ti/Si or CoMnN/Cu/Ti/Si thin films) in a home-made electrolytic cell using an external Agilent B2902A power supply, as indicated in earlier works. The electrolyte consisted of anhydrous propylene carbonate with Na^+^- and OH^–^-solvated species (10–25 ppm), in which metallic sodium was immersed to react with any possible trace of water. Negative voltages in this work signify the accumulation of negative charges at the working electrode (and vice versa for positive voltages). The magnetization (M) is obtained by normalizing the magnetic moment to the sample volume exposed to the electrolyte. Note that the linear slopes in the hysteresis loops at high fields (arising from diamagnetic or paramagnetic contributions) were eliminated by correcting the background signal (*i.e.*, at fields always significantly larger than the saturation fields).

### Structural and Compositional Measurements

The θ/2θ X-ray diffraction (XRD) patterns were collected on a materials research diffractometer (MRD) from Malvern PANalytical company, equipped with a PIXcel^1D^ detector, using Cu Kα radiation. High-resolution transmission electron microscopy (HRTEM), high-angle annular dark-field scanning transmission electron microscopy (HAADF-STEM), and electron energy loss spectroscopy (EELS) were carried out on a TECNAI F20 HRTEM/STEM microscope operated at 200 kV. Cross-sectional lamellae were prepared by a focused ion beam, placed onto a copper transmission electron microscopy grid, and topped with a protective platinum layer.

### Transport Measurements

To determine the electrical properties of CoN and CoMnN thin films, both films were deposited directly onto high-resistivity Si substrates. Resistivity values were recorded from 30 to 300 K by utilizing the 4-contact van der Pauw configuration in a closed He refrigeration system.

### *Ab initio* Calculations

First-principles calculations were based on the projector-augmented wave (PAW)^51^ method as implemented in the VASP package^52^ using the generalized gradient approximation.^53^ To model a Mn-doped Co and compare it to bare Co, we used (4 × 2) supercells with a four-monolayer thickness for both Co(90%)Mn(10%) and Co. To evaluate the energy barrier a N atom needs to overcome in order to be inserted in the surface, the nudged elastic band method (NEB)^49,50^ was used on the nitrogen pathway. At each step, the atomic coordinates were relaxed until the forces became as small as 1 meV Å^–1^. A kinetic energy cutoff of 500 eV was applied for the plane-wave basis set and 25 × 25 × 1 *k*-point meshes were used.

## Supporting Information Available

Dependence of the carrier densities (donor/acceptor) of as-prepared CoMnN films as a function of temperature; magnetoelectric characterizations for CoN and CoMnN films; structural characterization by HRTEM; and compositional characterization by TEM and energy-dispersive X-ray analysis (EDX) line scans (PDF)
