# Supplementary material for: From Binary to Ternary Transition-Metal Nitrides: A Boost toward Nitrogen Magneto-Ionics
Source: ACS Appl Mater Interfaces. 2022 Sep 21;14(39):44581–90. doi: 10.1021/acsami.2c12847 (PMC9542705; doi:10.1021/acsami.2c12847)
Supplement: Supplementary file 1 — am2c12847_si_001.pdf [file am2c12847_si_001.pdf]

Supporting Information

From Binary to Ternary Transition Metal Nitrides: A Boost toward Nitrogen Magneto-Ionics

Zhengwei Tan[1], *Sofia Martins*[1], *Michael Escobar*[1], *Julius de Rojas*[1,2], *Fatima Ibrahim*[3], *Mairbek Chshiev*[3,4], *Alberto Quintana*[5], *Aitor Lopeandia*[1,6], *José L. Costa-Krämer*[7], *Enric Menéndez*[1,*] *and Jordi Sort*[1,8,*]

[1]Departament de Física, Universitat Autònoma de Barcelona, E-08193 Cerdanyola del Vallès, Spain

[2]Department of Physics, Durham University, South Rd., Durham DH1 3LE, United Kingdom

[3]Univ. Grenoble Alpes, CEA, CNRS, SPINTEC, 38000 Grenoble, France

[4]Institut Universitaire de France, 75231 Paris, France

[5]Institut de Ciència de Materials de Barcelona (ICMAB-CSIC), Campus UAB, Bellaterra, E-08193 Barcelona, Spain

[6]Catalan Institute of Nanoscience and Nanotechnology (ICN2), CSIC and BIST, Campus UAB, Cerdanyola del Vallès, E-08193 Barcelona, Spain

[7]IMN-Instituto de Micro y Nanotecnología (CNM-CSIC), Isaac Newton 8, PTM, 28760 Tres Cantos, Madrid, Spain

[8]Institució Catalana de Recerca i Estudis Avançats (ICREA), Pg. Lluís Companys 23, E-08010 Barcelona, Spain

*Corresponding authors: E. Menéndez (Email: enric.menendez@uab.cat) and J. Sort (jordi.sort@uab.cat)

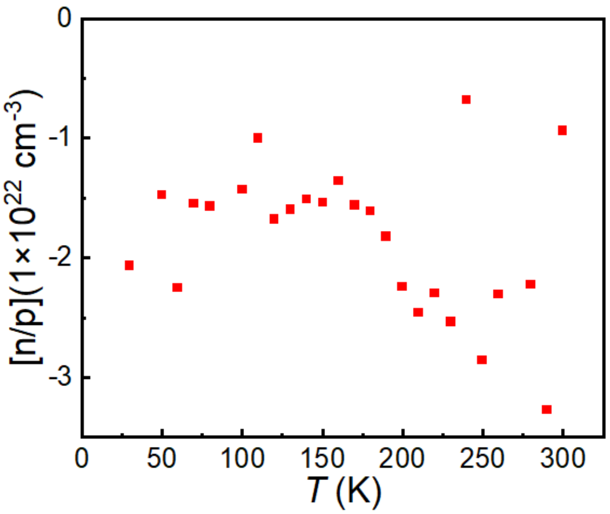

Figure S1. Dependence of the carrier densities (donor/acceptor) of as-prepared CoMnN films as a function of temperature.

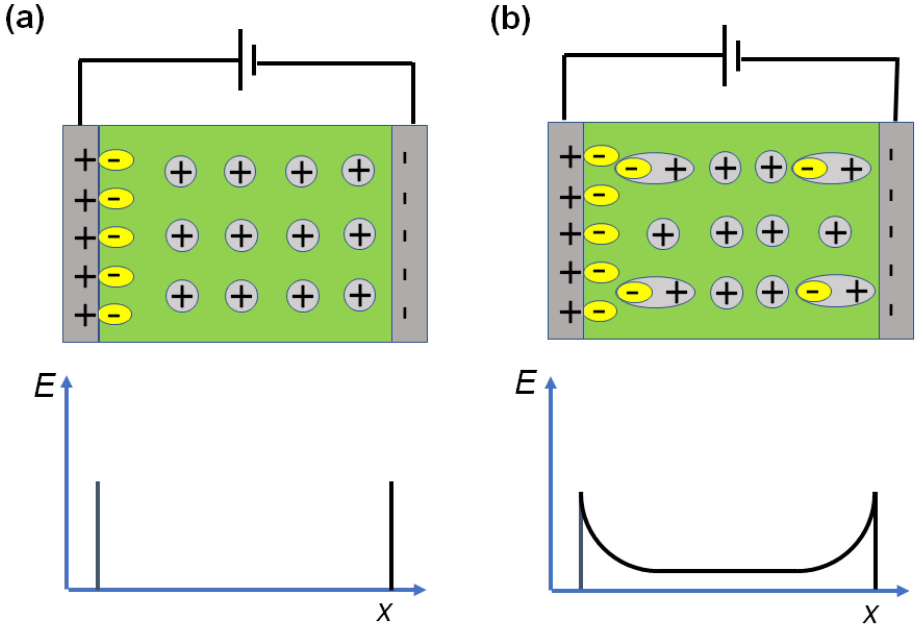

Figure S2. Cartoon representing electric penetration in a metallic (a) and a semiconducting (b) film based on the results of CoN films with dissimilar microstructure and electric properties as reported in reference 25. *x* represents the length across the film.

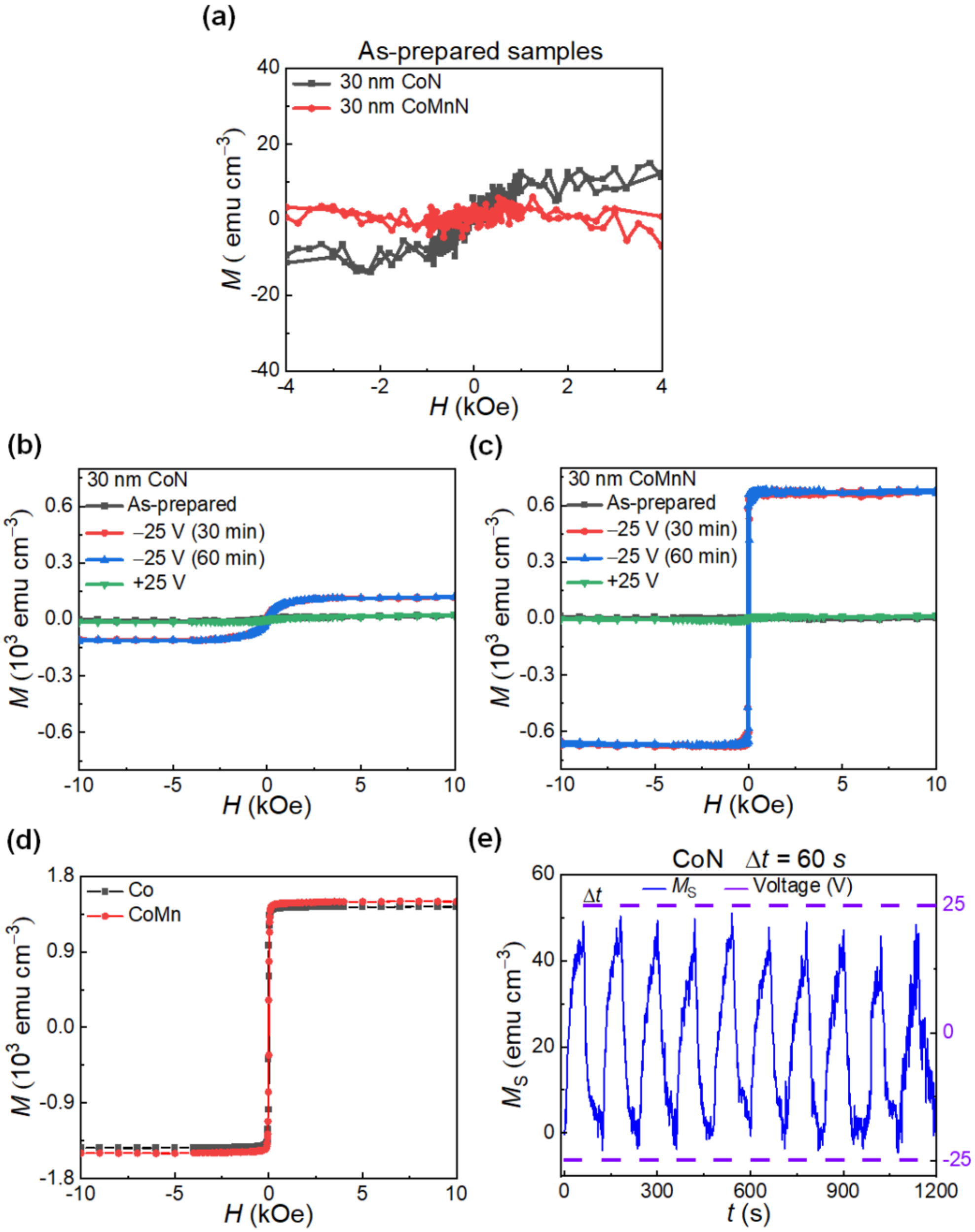

Figure S3. Magnetoelectric characterization of CoN and CoMnN films by in-plane vibrating sample magnetometry (each hysteresis loops of 30 min of duration) while electrolyte-gating. (a) Hysteresis loops of the as-prepared CoN and CoMnN films. (b) and (c) Hysteresis loops of the as-prepared film (black), while gating at –25 V for 60 min (red, 1st loop, and blue, 2nd loop) and after subsequent +25 V gating (green) for the CoN and CoMnN films, respectively. (d) Hysteresis loops corresponding to Co and CoMn (10 at. % Mn) films prepared in the same fashion as the nitrides but in vacuum and, thus, without nitrogen. (e) Magneto-ionic cyclability of the CoN films subjected to –25V/+25 V for 1 min at each voltage.

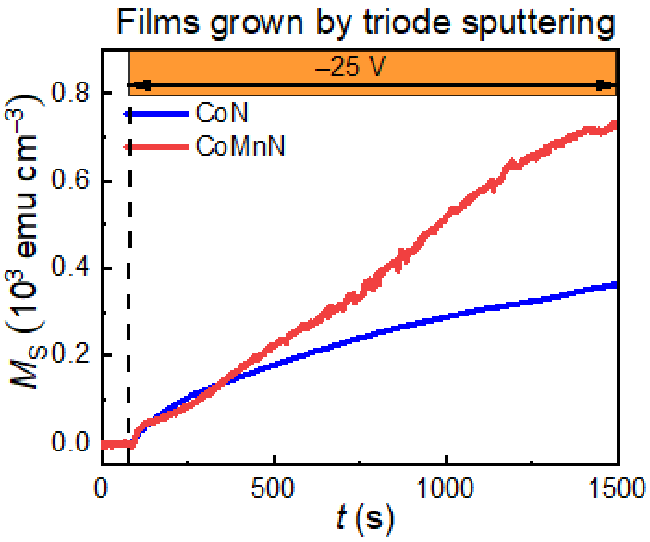

Figure S4. Time evolution of the saturation magnetization (M_S *vs.* t) of 85 nm-thick CoN and CoMnN films grown by homemade triode sputtering under electrolyte-gating at –25 V.

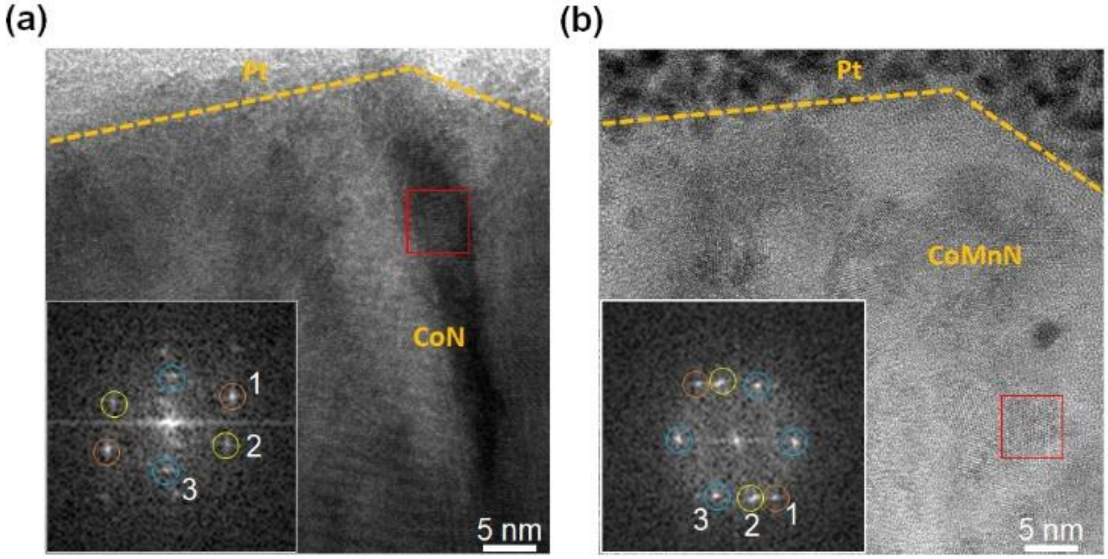

Figure S5. Structural characterization by High resolution transmission electron microscopy (HRTEM). (a), (b) HRTEM images of the cross section of CoN and CoMnN films after voltage treated upon –25V for 40 minutes. The inset shows the fast Fourier transform (FFT) of the area marked with a red rectangle. For phase identification, the cards no. ICDD JCPDF 00-001-1277, ICDD JCPDF 00-009-0418 and ICDD JCPDF 01-078-1991 were taken for Co, Co_3O_4 and $Co_{0.9}Mn_{0.1}O$, respectively. Pt layers serve as protective capping layer for lamellae preparation.

Spot label	*Interplanar distance d (Å)*	*(h k l) planes*
Figure S5 (a)-CoN		
1	2.06	(0 0 2) Co
2	2.19	(1 0 0) Co
3	2.41	(3 1 1) Co_3O_4
Figure S5 (b)-CoMnN		
1	2.18	(1 0 0) Co
2	2.43	(3 1 1) Co_3O_4
3	2.47	(1 0 0) $Co_{0.9}Mn_{0.1}O$

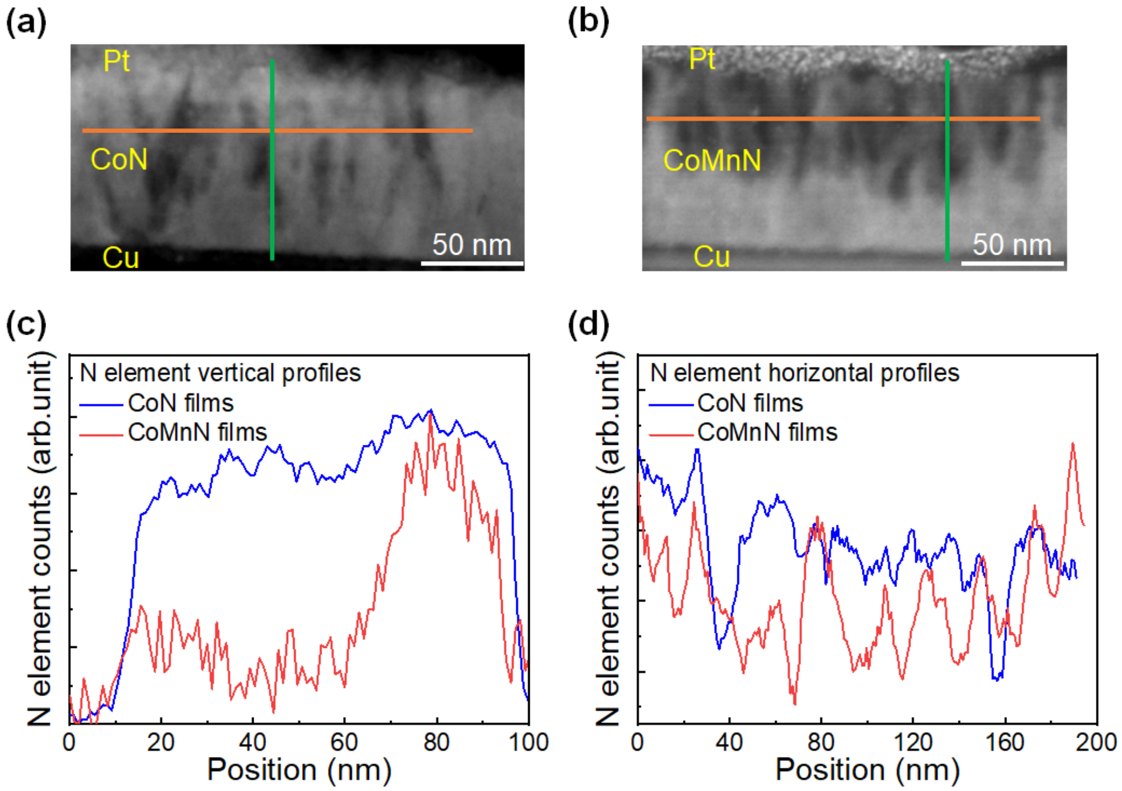

Figure S6. Compositional characterization by TEM and EDX line scans. (a) (b) TEM areas chosen for N element detection along depth (green line) and along horizontal (orange line) profiles for CoN and CoMnN films, respectively. (c) (d) are the N element line scans as a function of displacement along the vertical (depth) and horizontal directions.
